# Spatiotemporal assessment of post-harvest mycotoxin contamination in rural North Indian food systems

**DOI:** 10.1016/j.foodcont.2021.108071

**Published:** 2021-08

**Authors:** Anthony J. Wenndt, Hari Kishan Sudini, Rukshan Mehta, Prabhu Pingali, Rebecca Nelson

**Affiliations:** a School of Integrative Plant Sciences, Cornell University, Ithaca, NY, USA; b International Crops Research Institute for the Semi-Arid Tropics, Patancheru, Telangana, India; c Tata Cornell Institute for Agriculture & Nutrition, Cornell University, Ithaca, NY, USA; d Nutrition & Health Sciences, Rollins School of Public Health, Emory University, Atlanta, GA, USA; e Charles H. Dyson School of Applied Economics & Management, Cornell University, Ithaca, NY, USA

**Keywords:** Aflatoxin, Fumonisin, Deoxynivalenol, Post-harvest surveillance, Smallholder food systems

## Abstract

The spatiotemporal trends in aflatoxin B1 (AFB1), fumonisin B1 (FB1), and deoxynivalenol (DON) accumulation were analyzed in a range of food commodities (maize, groundnut, pearl millet, rice, and wheat) in village settings in Unnao, Uttar Pradesh, India. Samples (n = 1549) were collected across six communities and six time points spanning a calendar year and were analyzed for mycotoxins using enzyme-linked immunosorbent assays. AFB1 and FB1 were common across surveyed villages, with moderate to high detection rates (45–75%) observed across commodities. AFB1 levels in maize and groundnuts and FB1 levels in maize and pearl millet frequently exceeded regulatory threshold levels of 15 μg/kg (AFB1) and 2 μg/g (FB1). DON was analyzed in wheat, with 3% of samples yielding detectable levels and none exceeding 1 μg/g. In rice, AFB1 levels were highest in the bran and husk and lower in the kernel. Commodity type significantly influenced AFB1 detection status, while commodity type, season, and visual quality influenced samples’ legal status. Storage characteristics and household socioeconomic status indicators did not have significant effects on contamination. No significant effects of any variables on FB1 detection or legal status were observed. Data on mycotoxin contamination, combined with data on local dietary intake, were used to estimate spatiotemporal mycotoxin exposure profiles. Estimated seasonal *per capita* exposure levels for AFB1 (5.4–39.3 ng/kg body weight/day) and FB1 (~0–2.4 μg/kg body weight/day) exceeded provisional maximum tolerable daily intake levels (1 ng/kg body weight/day for AFB1 and 2 μg/kg body weight/day for FB1) in some seasons and locations. This study demonstrates substantial dietary mycotoxin exposure risk in Unnao food systems and serves as an evidentiary foundation for participatory food safety intervention in the region.

## Highlights

• Aflatoxin B1 and fumonisin B1 are prevalent in the food systems, while deoxynivalenol is largely absent.
• Mycotoxin contamination in Indian villages has distinct spatial and seasonal trends.
• Food system composition and cropping calendars influence the distribution of mycotoxins.
• Trends in contamination are associated with dietary intakes at levels that warrant public health intervention.

## 1 Introduction

Mycotoxins such as aflatoxins, fumonisins, and trichothecenes in food and feed are associated with a range of negative health and nutrition outcomes (Claeys et al., 2020; Khlangwiset et al., 2011). In most low-resource contexts, regulatory systems lack the ability to detect and remove mycotoxin-contaminated products from the supply chain (Wagacha & Muthomi, 2008). This is especially true in production systems dominated by smallholder farmers, where food products are typically consumed without quality screening. In India, poor resource access, literacy deficits, and credit constraints limit the ability of smallholders to produce food that complies with food safety regulations (Umali-Deininger & Sur, 2007). Smallholder farming communities in the region remain underregulated and ill-equipped to assess and manage food safety risk factors (Kumar & Popat, 2010; Mudili et al., 2014).

Mycotoxin surveys from India have yielded evidence of widespread food system contamination and have revealed some key drivers of exposure risk (Bhat et al., 1997; Priyanka et al., 2014; Reddy et al., 2000; Toteja et al., 2006). Much has been learned from these efforts, and they have provided a basis for significant concern. However, because conventional surveys of contamination status provide only fixed snapshots, they give limited insight into the nature of a dynamic problem that is suspected to vary considerably over time and space. Geography, climate, and cropping patterns, combined with pre- and post-harvest crop management practices, all play important roles in shaping patterns of mycotoxin accumulation (Wu & Khlangwiset, 2010). Consideration of the temporal and spatial distributions of these features within food systems is essential for targeting intervention options that best meet local needs.

A community's risk profile is determined both by pre- and post-harvest drivers of mycotoxin accumulation, and understanding the respective influence of each is an important aspect of effective management (Torres et al., 2014; Udomkun et al., 2017). Several pre-harvest factors influence crops' vulnerability to mycotoxigenic fungi in the field, including soil moisture (influenced by precipitation and soil organic matter content), fertilizer regimes, and varietal traits (Craufurd et al., 2006; Manoza et al., 2017; Mutiga et al., 2017). Some commodities are more vulnerable to mycotoxin accumulation in the field than others, and there can be marked year-to-year variability in contamination outcomes, largely associated with climatic phenomena (Afsah-Hejri et al., 2013; Mitchell et al., 2016). Features of agro-ecological zones are known to be associated with population-level nutrition outcomes, which may be linked to differential levels of mycotoxin exposure across environments (Mutegi et al., 2009; Smith et al., 2012).

Post-harvest mycotoxin accumulation resulting from poor storage conditions also influences the overall toxin burden (Hell et al., 2000; Villers, 2014). A range of possible factors in the post-harvest ecology may give rise to increased risk of mycotoxin accumulation, including high initial crop fungal contamination, poor quality/under-maintained storage containers, excessive moisture or heat in the storage environment, abundance of fungus-vectoring pests, and others. The vulnerability of indigenous grain storage structures to spoilage phenomena and mycotoxin accumulation in numerous crops has been reported in the literature (Sashidhar et al., 1992; Tefera et al., 2011). Profiling crops’ initial toxin loads upon entry into the storage environment, and the subsequent accumulation of toxins over the course of storage time, can elucidate the relative importance of pre-versus post-harvest factors in determining the spatiotemporal distributions of contamination and exposure risk.

Because mycotoxins are invisible and expensive to measure, resource-poor communities typically lack awareness of and access to the range of possible mitigation options. Participatory research is one strategy for bolstering problem-solving capacity in low-resource settings by engaging directly with farmers in the research process (Nelson et al., 2001; Omanya et al., 2007; Trimble & Berkes, 2013). The present study is part of an on-going effort by a consortium of partners under the Technical Assistance and Research for Indian Nutrition and Agriculture (TARINA) program, spearheaded by the Tata-Cornell Institute for Agriculture and Nutrition, which aims to leverage participatory research to enable vulnerable populations to monitor and address food safety challenges that constrain food security, community health, and nutrition.

In this study, we sought to develop an understanding of the drivers of mycotoxin contamination and exposure risk in Unnao District, Uttar Pradesh, which would serve as an evidentiary foundation for participatory food safety intervention in the region. Concurrent with ongoing participatory programs in targeted communities, a longitudinal survey of the accumulation of three major mycotoxins (aflatoxin B1, fumonisin B1, and deoxynivalenol) was conducted in household stores of rice (unmilled), wheat, maize, groundnut, and pearl millet. As it is known that the aflatoxins in rice differentially accumulate across husk, bran, and endosperm tissues, the extent to which the aflatoxin burden in stored rice was distributed across these tissues was also investigated. Evidence for and against several key risk factors as determinants of toxicity status in these smallholder food systems is presented along with estimates of seasonal dietary mycotoxin exposure levels in the target population based on local diets. It is determined that the dietary mycotoxin burden in rural Unnao District is sufficient to warrant the development and evaluation of a participatory intervention approach.

## 2 Materials & methods

### 2.1 Longitudinal survey sites and selection criteria

The site selection process commenced in September 2017. Several selection criteria were used to screen candidate areas, including: 1) predominantly rural with majority smallholder (<2 ha) farmers, 2) historically disadvantaged in terms of socioeconomic status, and 3) demonstrable risk of dietary mycotoxin exposure. Unnao District arose as a suitable location after consultation with consortium partners. The district is situated in the Indo-Gangetic Plain region of northern India, bordering the Ganges River to the West. Unnao encompasses a total area of 4558 mi^2^ and has a population of 3,108,367 as of the 2011 census (Census of India, 2011). Average annual rainfall in Unnao totals 852 mm, with average temperatures ranging from 19.3 to 32.2 C (Singh, 2013). Mean annual rainfall in Unnao ranged from 187 to 507 mm across locations Unnao locations in 2017 (Ansari, 2019). There are distinct rainy (*kharif*) and post-rainy (*rabi*) seasons in the region that vary greatly in terms of temperature and rainfall.

Initial assessments prompted the delineation of two food system types that generally corresponded to block-level administrative boundaries and feature distinct trends in cropping system composition and timing (Fig. 1b and c). Wheat was the predominant *rabi* crop in both systems. *Kharif* production in Hasanganj block was largely rice-based, while in Bangarmau block, farmers grew rice along with maize, groundnut, and pearl millet in this season. Grain and legume production were less common in the summer *zaid* season, but there was some cultivation of maize and groundnut, especially in Bangarmau block.

**Fig. 1:**
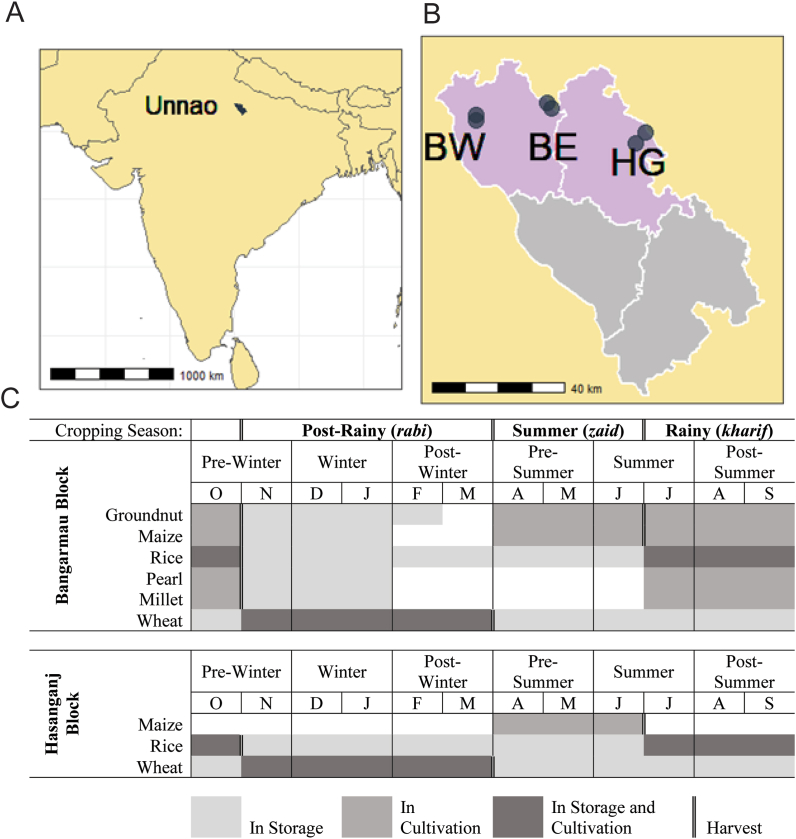
(A) Position of Unnao District within India. (B) Map of Unnao District indicating villages participating in the longitudinal survey. Pink shaded regions correspond to the Hasanganj (right) and Bangarmau/Safipur (left) administrative *tehsils*. BW = Bangarmau-West village cluster, BE = Bangarmau-East village cluster, HG = Hasanganj village cluster. (C) Cropping systems calendar for the distinct ‘diverse *kharif*’ (Bangarmau) and ‘rice *kharif*’ (Hasanganj) cropping systems. Maps in panels (A) and (B) were created using a shapefile from the public domain Natural Earth database (naturalearthdata.com). (For interpretation of the references to color in this figure legend, the reader is referred to the Web version of this article.)

In the 2011 Indian census, the district population was 83% rural and had an average literacy rate of 66%, which is less than both the state (68%) and national (74%) figures (Census of India, 2011). Among Unnao's rural population, an estimated 22–24% live below the poverty line Chaudhuri and Gupta, 2009, World Bank, 2010. These findings satisfied the first two inclusion criteria. In an earlier survey of marketplaces in Lucknow City, located adjacent to Unnao District, 78% of the 32 maize samples tested had detectable aflatoxin and the mean concentration was above the Indian regulatory legal limit of 15 μg/kg, satisfying the third inclusion criterion (Chandra et al., 2013).

Based on the reported findings from Lucknow, ≥50% prevalence and 15 μg/kg mean contamination of maize and groundnut samples were used as village-level inclusion criteria. Only villages with demonstrable mycotoxin burdens were considered, as this study was intended to inform subsequent intervention activities in high-risk communities. Out of twelve villages identified as candidates from initial consultations with local extension officials, six were selected for the study and their eligibility based on contamination status was confirmed after the first sampling time point. Two villages were located in wheat- and rice-dominated Hasanganj and constituted “lower risk” sites based on the low prevalence of aflatoxin- and fumonisin-susceptible commodities. The remaining four villages in Bangarmau block, which had relatively diverse *kharif* crops, constituted “higher risk” food systems. Based on spatial and sociocultural proximities, we grouped the six villages into three “clusters”: Hasanganj, Bangarmau-East, and Bangarmau-West.

### 2.2 Longitudinal sampling and survey methodology

We conducted a longitudinal survey with time-series sampling of specific household grain storage units every two months for a full calendar year (six sampling time points from October 2017 onward), beginning in the Pre-Winter season (Fig. 1c). Farmer participants voluntarily enrolled their commodities for monitoring and were encouraged to engage in the surveillance process. Several commodity inclusion criteria were used, including 1) grain intended entirely or partially for household use as food, 2) units located in or near household premises, and 3) units intended/fit for long-term storage (i.e. not in temporary storage or intended for immediate use). Five types of stored commodities were enrolled: maize, groundnut, pearl millet, rice (unmilled), and wheat. During each household visit, ~50 g samples were drawn from each enrolled storage unit and deposited into a labelled sample pouch. Whenever possible, samples were extracted systematically by taking a single representative diagonal core with a sampling spear through an opening in the unit. In cases in which it was mechanically or culturally not appropriate to systematically sample, we sampled the next consumable fraction – practically, a deep handful drawn by the farmer participant from the storage unit at its most accessible point.

If the respondent consented to further probing during sampling, moisture content was recorded for each rice, wheat, maize, and pearl millet sample using a handheld grain moisture meter. Due to field equipment limitations, moisture data for groundnut samples was not collected. Each sampling event was accompanied by a brief questionnaire, in which the surveyor and farmer both were asked to evaluate quality parameters including a visual quality rating from “1” = terrible to “5” = excellent. Visual quality scores of the researcher and the farmer participant were averaged to arrive at a final rating that reflects both the farmers' intimate contextual knowledge and the surveyors’ formal scientific training. After collection, the grain samples were stored under refrigeration to prevent further microbial growth until being sent to the laboratory for processing and mycotoxin quantification.

### 2.3 Mycotoxin analysis

Each food sample was ground to a fine powder using a Kenstar Senator laboratory blender (Gurugram, India). For aflatoxin B1 (AFB1) and fumonisin B1 (FB1) extractions, 100 ml of 70% methanol (v/v-70 ml absolute methanol in 30 ml distilled water) containing 0.5% KCl was added to 20 g sample powder containing 0.5% KCl in an Erlenmeyer flask. For deoxynivalenol (DON) extractions, 100 ml diH_2_O was used in place of methanol, in accordance with the ELISA kit manufacturer's protocol. Extracts were incubated at room temperature for 60 min on a revolving shaker at 250 rpm, filtered through Whatman No. 4 filter paper into a fresh tube, then stored at 4 °C until analysis. A similar protocol was used to prepare a toxin-free sample extract from healthy groundnut, which was used for dilution of standards and as a negative control.

Indirect competitive ELISAs were performed to quantify AFB1 and FB1 according to published protocols developed by ICRISAT (Barna-Vetró et al., 2000; Waliyar & Sudini, 2012). The limit of detection (LOD) for the AFB1 assay was 0.1 μg/kg and limit of quantification (LOQ) was 1 μg/kg, with 93% recovery, as previously reported (Reddy et al., 2001). The LOD (7.6 μg/kg) and LOQ (10 μg/kg) for FB1 were determined by the assay developers. ELISA plates were coated with 150 μl AFB1-Bovine Serum Albumin (BSA) for AFB1 ELISAs or FB1-BSA for FB1 ELISAs, both prepared in carbonate buffer (100 ng/ml) and incubated at 37 °C for 1 h. Blocking was conducted by adding phosphate-buffered saline with Tween 20®-BSA (PBST-BSA) to each well and incubating at 37 °C for 30 min. AFB1 (range 25–0.097 ng/ml) and FB1 (range 6–0.05 μg/ml) standards were prepared in 10% toxin-free extract with 7% methanol and included in duplicate. Next, 100 μl of diluted sample extract (1:10 in PBST-BSA) and 50 μl of antiserum diluted in PBST-BSA (1:6000 for AFB1; 1:5000 for FB1) were added to all wells and incubated at 37 °C for 1 h. Incubation in secondary anti-body was conducted by adding 150 μl anti-rabbit-IgG alkaline phosphatase (IgG-ALP) (1:4000 in PBST-BSA) to all wells and incubating at 37 °C for 1 h. The ELISA plates were washed three times with PBST after each stage of incubation. Finally, the substrate solution was prepared by dissolving 15 mg *p*-nitrophenyl phosphate in 20 ml of 10% diethanolamine and added to each well. Plates were incubated at room temperature until color development (~20 min; no stop solution added), and absorbance was measured immediately at 405 nm using a Bio-Rad iMark microplate reader (Bio-Rad Laboratories, CA, USA).

For DON analysis, a commercially available test kit (Helica Biosystems, CA, USA) was used to perform direct competitive ELISAs. The assay was conducted as per the kit instructions and had an LOQ of 10 μg/kg as reported by the manufacturer. First, 200 μl of the conjugate solution was mixed with 100 μl sample extract or DON standard (10-0 μg/ml) in a 96-well dilution plate. After dilution, 100 μl of the contents from each dilution well was transferred to the corresponding antibody-coated microtiter well of the kit's test plate and incubated at room temperature for 15 min. The contents of the test plate were discarded, and the plate was washed 3 times with a PBST wash buffer. Subsequently, 100 μl of the substrate reagent was added to each well and incubated at room temperature for 5 min. Finally, 11 μl of stop solution was added to the plate in the same sequence as the substrate reagent. Absorbance was read at 450 nm using the same instrument as described above.

All samples were assayed in duplicate on the ELISA plates. Absorbance was recorded and processed using Microplate Manager 6 software (Bio-Rad Laboratories, CA, USA). Second-order polynomial standard curves were generated for each AFB1 and FB1 ELISA plate, plotting Log_10_ values of the standards against their absorbance values. For DON, standard curves were generated according to manufacturer's instructions by calculating % bound and plotting against the DON content of each standard. For all toxins, the standard curves were used to compute sample concentrations by interpolation, taking all sample dilution stages into account. Samples with absorbance values less than the highest concentration of the standard curve (25 ng/ml, 6 μg/ml, and 10 μg/ml for AFB1, FB1, and DON, respectively), were serially diluted and re-analyzed until their absorbance values were within the range of the standard curve. Aflatoxin and DON contamination values were compared to the Indian regulatory limits of 15 μg/kg and 1 μg/g, respectively. At present, the Indian government has not specified regulatory limits for FB1 in food, so the USA regulatory limits of 2 μg/g was used as a regulatory threshold in this study.

### 2.4 Rice milling status and componential AFB1 analysis

In order to determine how much of the toxin in unmilled rice is present in the polished rice intended for human consumption, paired samples of unmilled and milled rice from were collected from ~30 random households across the study sites at the fourth and sixth time points (n = 58 total pairs). Paired samples were processed for AFB1 analysis as described. The differential accumulation of AFB1 across rice husk, bran, and endosperm tissues was investigated using a small convenience sample of rice from mill facilities in peri-urban Lucknow, the metropolitan hub nearest to Unnao District. Duplicate samples of husk, bran, and kernel components were collected from the same milled batch at each of six milling facilities. Brief interviews were conducted with each mill operator based on a questionnaire regarding the fate and end usage of each component. Samples were processed and analyzed for AFB1 as described.

### 2.5 Food consumption data collection

A semi-quantitative food frequency questionnaire (FFQ) with monthly and daily recalls (including portion sizes) was used to estimate monthly and daily food frequencies and portion sizes for all 12 months, as adapted from an FFQ previously validated for the North Indian context (Telles et al., 2016). A total of 31 random households (~10 per village cluster) were selected from the three village clusters. Questionnaire-guided interviews were conducted with the member of the household primarily responsible for food preparation, who was usually female. Given the context of very conservative local gender dynamics and expressed hesitation about revealing details about their food security among respondents, household FFQ interviews were anonymized to respect respondents’ privacy and ensure comfort. First, each respondent was asked whether rice (polished), wheat (flour), maize (fresh or flour), pearl millet (flour), and groundnut (fresh/roasted) were consumed in the household at any point throughout the year. For each affirmative response, the respondent was asked to report the following:

1. In which months is this commodity consumed?
2. In each month, how many people in the household are eating this product when it is prepared?
3. In each month, how frequently is this food consumed?
4. In each month, how much of this food (g) is used in a single meal/preparation?

To precisely estimate portion sizes, respondents were asked to produce the vessel (glass, bowl, etc.) typically used to measure the grain or flour. Then they were asked to fill the vessel to the level they would use for a single meal/preparation of that food item for the household. The quantity was weighed using a portable electronic balance. If groundnut portion sizes were computed using groundnuts in shells, masses were adjusted using the shelling percentage of 65%, as estimated for the groundnut varieties grown in the region (Upadhyaya et al., 2012; Varshney et al., 2014).

### 2.6 Dietary exposure estimation

Commodity-wise mycotoxin intakes were estimated deterministically using a standard exposure dose calculation method (ATSDR, 2005). The formula used was:

Dose = [mycotoxin (μg/kg)] x [daily consumption (kg)]/body weight (kg).

where the concentration of mycotoxin was the mean level for that commodity at the respective locality and season, and the daily consumption level was the average *per capita* daily grain intake in a given locality and month. For estimates of AFB1 exposure from rice, we used contamination data from polished rice analyzed in the paired milled/unmilled rice sampling at each locality as described above. As we did not have data on the ratio of children to adults in the surveyed homes, we computed household *per capita* body weight using existing demographic data from the region. Jha et al. (2008) used values of 70 kg and 20 kg for adults and children, respectively, for exposure dose estimation in Unnao District. On average, 34.7% of the population in rural Uttar Pradesh is under 14 years of age (Census of India, 2011). Thus, we multiplied 34.7% of the total number of consumers in each household by 20 kg and the remaining 65.3% of the number of consumers by 70 kg, then summed the two to arrive at a *per capita* body weight estimate of 52.7 kg. Unique *per capita* exposure dose estimations were made for each household, commodity, and month across all four village clusters, allowing us to assess seasonal fluctuations in dietary mycotoxin intake and to explore the relationships between food grain contamination and exposure risk.

While no formal tolerable daily intake level is currently specified for AFB1, a provisional maximum tolerable daily intake (PMTDI) level of 1 ng/kg body weight/day has been previously suggested (Kuiper-Goodman, 1998). The World Health Organization (WHO) has issued a PMTDI of 2 μg/kg body weight/day for FB1 (World Health Organization, 2002). These PMTDI thresholds were used in this study to interpret risk levels for dietary exposure.

### 2.7 Statistical analysis

Pearson's correlations between farmers' and surveyors' grain quality ratings were computed for each commodity type to assess the consistency of quality scores. One-way analysis of variance (ANOVA) with post-hoc Tukey HSD tests for multiple comparisons were used to assess differences in moisture content across sampled commodities and seasons. Mycotoxin detection and regulatory legal status were expressed as binary and compared across village clusters, commodity groups, and time points. Mean values of AFB1, FB1, and DON for each sample were transformed to log_10_ (x+1) to normalize the distribution of the data in analyses of contamination levels. Pre- and post-processing rice pairs were compared using paired one-sided t-tests. Descriptive statistics were generated using R (version 3.6.1, 2019), and correlation matrices were used to check for multicollinearity among variables prior to modeling. Shapiro-Wilk normality tests indicated non-normal distributions of mycotoxin levels. Due to the highly skewed nature of these data, we coded toxin detection and legal status as binary response variables (Y/N) according to the LOQs specified above and the legal regulatory limits of 15 μg/kg, 2 μg/g, and 1 μg/g for AFB1, FB1, and DON, respectively.

To test whether variability in toxin outcomes could be explained by seasonal dynamics, storage conditions, or household-level characteristics, generalized linear mixed models (GLMM) for binomial distributions and logit link functions were used for multivariate analysis. The models were performed using the glmer function in the R package ‘lme4’ (Bates et al., 2015). GLMM is a robust strategy for modeling data with hierarchical/clustered organization, repeated measures, and imbalanced samples sizes (Kutt & Gordon, 2012). In mixed effects models, inclusion of random effects is used to account for non-independence of observations caused by clustering or hierarchical structuring of data. For AFB1 and FB1, models of 1) toxin detection and 2) toxin legal status were constructed. Due to rareness of DON contamination observed in our study, it was not possible to construct models for this toxin class.

Sample-level fixed effects in the AFB1 models included season [six levels; pre-winter (reference) – post-summer], commodity type [four levels; rice (reference), maize, groundnut, and pearl millet], duration of storage time (d), container type [four levels; jute sack (reference), polypropylene sack, other (modern), and other (traditional)], and average visual quality score (1–5 rating scale). Due to high incidence of missing moisture data (30%; including 100% of groundnut samples), it was not possible to include this variable in the models. The household-level fixed effects of socioeconomic status indicators, which have been shown to be associated with mycotoxin outcomes (Jolly et al., 2015; Leroy et al., 2015), were also examined. Landholding quartile (within villages) [four levels; low (reference), low-middle, upper-middle, and upper] and the percentage of wage-earning household residents were included as fixed effects representing stable household wealth. Variance components of several clustering variables that could be modeled as random effects, including household, nested in village, nested in village cluster, were examined. The cluster and village factors explained no variance and did not improve model fit compared to the inclusion of only a household-level random effect. ANOVA F-test and Akaike information criteria were used to confirm that the model with only household-level random effect was most parsimonious.

Similar models were constructed for FB1 detection and illegal status. As fixed effects, season, storage time, quality score, landholding quartile, and percent wage-earning residents were retained as above. For the fixed effect of commodity, only two levels were considered: millet (reference) and maize. Storage structures were not distributed evenly across FB1 outcome groups, and therefore this effect could not be modeled. Because adequate sample sizes for the two commodities were only achieved at pre-winter and winter sampling time points, it was not possible to model season as a fixed effect. Instead, season was included as a random effect to account for non-independence of observations within seasons, as described previously (Iwachido et al., 2020). Household was also included as a random effect. Model fit and parsimony diagnostics were performed as described above.

A linear mixed modeling (LMM) approach was used for analysis of AFB1 and FB1 dietary exposure levels. Similar to GLMM, this multi-level model incorporated random effects to account for non-independence of structured data sets (Millar & Anderson, 2004; Pinheiro & Bates, 2001). Cumulative *per capita* daily mycotoxin intake (ng/kg body weight/day for AFB1 and μg/kg body weight/day for FB1) was used as a continuous response variable. Seasonal variation in exposure was included as the major fixed effect of interest in the models. We included the fixed effect of household size (number of residents) to control for household socioeconomic status. Due to the anonymous nature of the FFQ data, there was limited knowledge of household characteristics and thus is was not possible to include other household-level fixed effects. The random effects of household nested in village and village cluster were included to account for the hierarchical organization of the data. Normal distribution of model residuals was confirmed by visualizing Q-Q plots using the qqmath function using the ‘lattice’ package in R (Sarkar, 2008). Model fit and parsimony diagnostics were performed as described above.

## 3 Results

### 3.1 Longitudinal survey sampling effort and food system composition

**Fig. 2:**
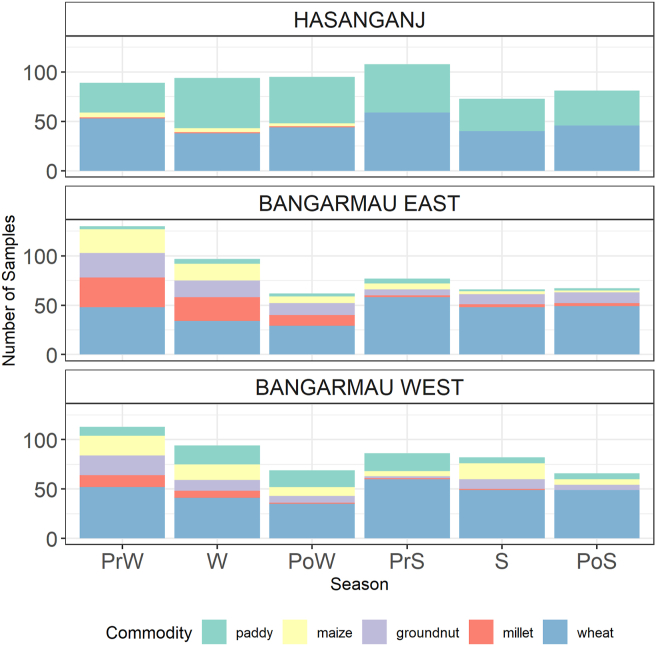
Summary of sample yields across seasons, by village cluster. Seasons: PrW = pre-winter; W = winter; PoW = post-winter; PrS = pre-summer; S = summer; and PoS = Post-Summer.

All available food grains in participating households were sampled at each time point, and therefore the sample yield serves as an indicator of seasonal dynamics in food system composition. In total, 1549 samples of wheat (832), rice (340), maize (143), groundnut (136), and pearl millet (98) were collected across the three village clusters and six time points, with marked variation in commodity availabilities over space and time (Fig. 2). Wheat was consistently the most abundant commodity in all village clusters and constituted the highest proportion of sample yield at every time point. The ubiquitous presence of wheat reflects the district-wide predominance as a post-rainy (*rabi*) growing season crop.

The other commodities, which were typically cultivated in the rainy (*kharif*) growing season, had more localized availabilities than wheat across village clusters and seasons. In Hasanganj, rice was the major (and nearly only) *kharif* season crop and was collected along with wheat in most households. The two clusters in Bangarmau block yielded much greater diversity in *kharif* season crops, owing to the relative abundance of maize, pearl millet, and groundnut cultivation in this area (Fig. 2). There was a distinctive seasonal peak in the availability of maize, groundnut, and pearl millet during the pre-winter and winter seasons, which corresponded both to local cropping calendars and to widespread cultural preference for these crops as winter foods. A small uptick was observed in maize during the summer, owing to a minor summer (*zaid*) cultivation, which was put into storage during this season. These findings suggest that food system composition is highly variable across time and across communities, even those in close spatial proximity.

### 3.2 Storage environments and grain quality

Typically, a local household's long-term grain storage facility consisted of a stack of 50–60 kg sacks, insulated above and below by finely chopped rice straw, *busa*, which is periodically used as livestock fodder. The most common storage container for all commodities was plastic sacks, constituting 62%, 75%, 71%, and 69% of all groundnut, maize, rice, and pearl millet storage systems, respectively. Of the remaining samples, most were stored in jute sacks, which were used in 19%, 9%, 14%, and 24% of groundnut, maize, rice, and pearl millet storage systems. Smaller proportions of grain stores constituted ‘other modern’ (e.g. metal drums, hard-sided containers, or packages) or ‘other traditional’ (e.g. mud/dung-plastered silos, woven baskets, earthen pots, etc.) storage systems. Maize was sometimes (8% of samples) stored in the open air, either shelled in piles or – more commonly – hung on the cobs from the ceiling. When stores diminished, typically after several months, many households transferred all grain types from sacks to smaller hard-sided containers such as plastic boxes, steel pots, or buckets.

Wheat was occasionally (12% of samples) stored in 500 kg-capacity metal drums. These drums were usually custom-built, and only used by wealthier farmers in villages where a metalsmith was manufacturing them. There is some evidence suggesting that these drums were effective in minimizing storage losses in South Asia (Alam et al., 2007). Preservative amendments to the storage environment were used in 16% of wheat stores. Neem leaves, salt, chemical powders, chemical bars, and matchbooks were used as preservatives in 9%, 4%, 3%, 3%, and 2% of wheat samples, respectively. The use of preservatives for non-wheat stores was very rare. Indigenous earthen storage structures, typically fashioned from bamboo, mud clay, brick, dung, and/or straw, were used rarely and only for storing rice. Farmers reported that these structures were being phased out of use in favor of sacks, which were considered easier to maintain and transport.

**Fig. 3:**
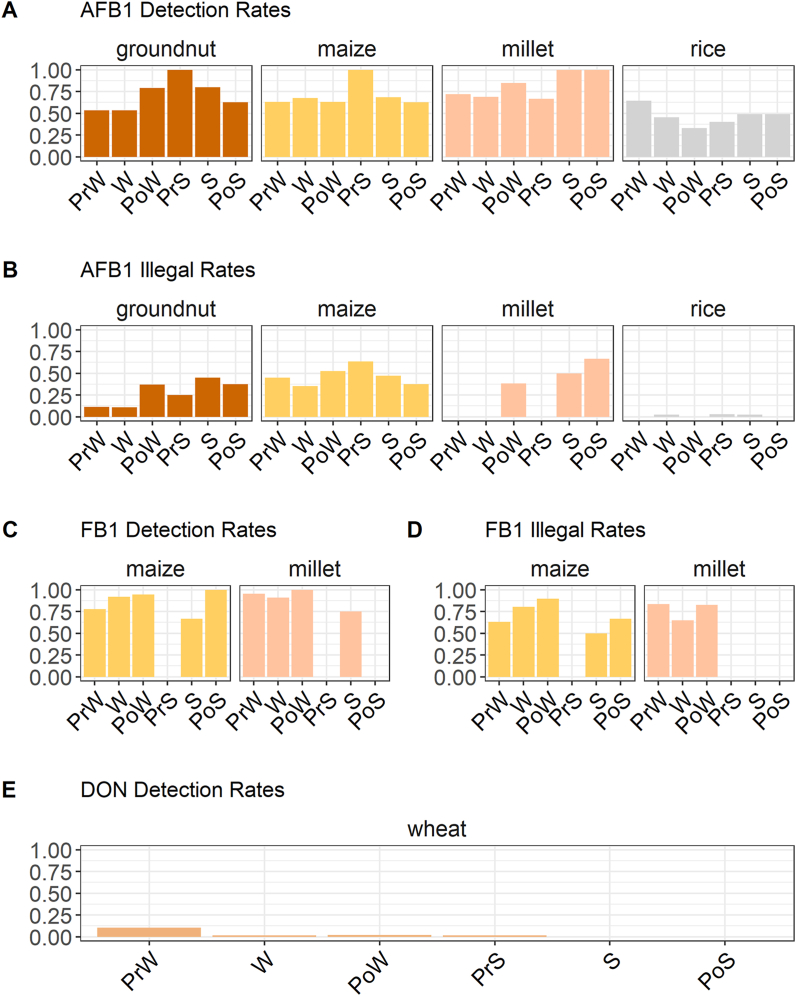
Detection and illegal status rates for all commodities and toxin classes across seasons. PrW = pre-winter, W = winter, PoW = post-winter, PrS = pre-summer, S = summer, PoS = post-summer. (A) Proportion of samples with detectable (>1 μg/kg) AFB1 across seasons, by commodity. (B) Proportion of samples with AFB1 exceeding 15 μg/kg across seasons, by commodity. (C) Proportion of maize and pearl millet samples with detectable (>10 μg/kg) fumonisin B1. (D) Proportion of maize and pearl millet samples with fumonisin B1 exceeding 2 μg/g, by commodity. (E) Proportion of wheat samples with detectable (>10 μg/kg) DON. No wheat samples exceeded the 1 μg/g regulatory limit for DON.

Mean quality scores were 3.77 (SE = 0.02), 3.56 (SE = 0.04), 3.46 (SE = 0.05), 2.58 (SE = 0.05), and 3.50 (SE = 0.02) for rice, maize, groundnut, and pearl millet, and wheat, respectively. There were modest yet statistically significant Pearson's correlations between farmers' and surveyors' scores for each commodity (Fig. S1), suggesting reasonable consistency of quality assessment. In total, grain moisture data were available for 66%, 28%, 96%, and 87% of maize, rice, pearl millet, and wheat samples, respectively. Grain moisture contents in storage ranged from 9.0 to 18.5%, with means between 11.7 and 12.3% depending on commodity type (Table 1). There were no significant differences in grain storage moisture across commodities (p > 0.05). In wheat and maize, moisture content dropped significantly between pre-winter (TP1) and winter (TP2), but there were no other significant differences between seasons. In rice, moisture content was significantly higher than average (p < 0.0001) at pre-winter, and significantly lower than average in pre-summer and summer (Fig. 3b). Pearl millet samples had significantly higher moisture content than average in pre-winter, and significantly lower moisture in post-winter (p < 0.01). In general, the mean moisture levels observed across all commodity groups and time points were sufficiently low to make excess microbial contamination unlikely (Walker et al., 2018; Yusuf & Yong, 2011).

**Table 1:** Summary of storage moisture contents (wet basis) across commodity types. Means represent equilibrium moisture content (%) in storage units across all locations and time points.

Commodity^a^	N	Mean	Minimum	Maximum	Standard Deviation
Maize	143	12.3	10.0	16.5	1.0
Pearl Millet	98	11.9	9.0	16.0	1.4
Rice	340	11.7	9.1	15.1	1.2
Wheat	725	12.0	9.4	18.5	0.8

a Moisture data were not collected for groundnuts due to instrumental limitations in the field.

### 3.3 Mycotoxin contamination status

Stored grains were frequently contaminated with aflatoxin B1 (AFB1) and fumonisin B1 (FB1) throughout the study area. AFB1 was detected in 75%, 68%, 65%, and 45% of pearl millet, maize, groundnut, and unmilled rice samples, respectively. Mean AFB1 contamination levels in samples with detectable toxin were 312 (range 1.1–8571.6), 233.6 (range 1.0–1864.3), 7.2 (range 1.1–61.4), and 4.4 (range 1.0–26.3) μg/kg for groundnut, maize, pearl millet, and rice, respectively.

**Fig. 4:**
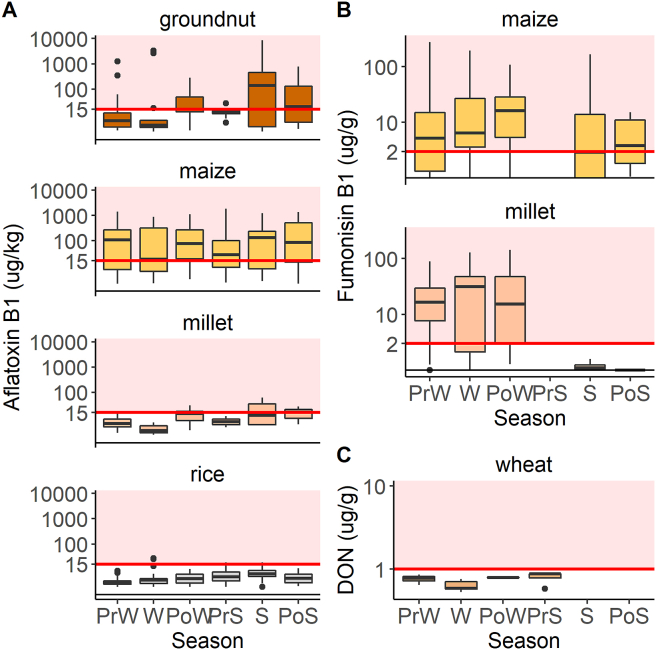
Season-wise mycotoxin contamination levels among samples with detectable levels of AFB1, FB1, and DON. Seasons: PrW = pre-winter, W = winter, PoW = post-winter, PrS = pre-summer, S = summer, PoS = post-summer. Data are plotted on log_10_ scales, with axis values representing actual (non-transformed) toxin concentrations. (A) AFB1 levels (μg/kg) in stored samples of groundnut, maize, pearl millet, and rice, (B) FB1 levels (μg/g) in stored samples of maize and pearl millet, (C) DON levels (μg/kg) in stored wheat samples. Red shaded regions correspond to levels above regulated legal maxima of 15 μg/kg, 2 μg/g, and 1 μg/g for AFB1, FB1, and DON, respectively. (For interpretation of the references to color in this figure legend, the reader is referred to the Web version of this article.)

Groundnut and maize samples had highest AFB1 detection rates in pre-summer, and illegal status (levels above the 15 μg/kg Indian legal limit) rates peaked in pre-summer and summer for groundnut and maize, respectively (Fig. 3). Although the AFB1 detection rate in rice was substantial across all time points, the magnitude of contamination rarely exceeded the legal limit (Fig. 4). The highest AFB1 detection rate in rice samples was observed during pre-winter (64%), but this decreased to 45% in the following season, by which time participating farmers had generally sold their surplus rice in the market. This suggests that, whether knowingly or not, farmers were preferentially discharging worse quality produce to the marketplace and keeping better quality produce for their own household consumption, as has been previously observed in other contexts (Hoffmann et al., 2013). High detection rates were also observed in pearl millet, but samples were generally contaminated at levels below the regulatory legal limit. In the summer and post-summer seasons, illegal status rates were high (50% and 67%, respectively) in pearl millet. However, sample sizes were very low (n < 5) at these time points, and so it is possible that these estimates are not representative.

No significant relationships between season and the odds of AFB1 detection (p = 0.61) were observed, reflecting the relatively uniform detection rates observed across seasons. On the other hand, seasonality had a strong significant effect on AFB1 legal status (p = 0.002; Table 2). Grain samples in winter and post-winter had significantly lower odds (OR 0.35–0.36, p = 0.001; Table S1) of AFB1 illegal status compared to the pre-winter reference level, while the summer season had significantly higher odds (OR 3.5, p = 0.03; Table S1). This trend is attributable to the observed increases in illegal status rates, predominantly in groundnut and pearl millet, in the later seasons (Fig. 3).

**Table 2:** Summary of GLMM results for AFB1 detection and illegal status in maize, groundnut, unmilled rice, and pearl millet samples. *P*-values in bold are significant at the p < 0.05 level.

	AFB1 Detection			AFB1 Illegal Status		
	χ^2^	Df	P	χ^2^	Df	P
(Intercept)	0.24	1	0.625	1.01	1	0.314
Season	2.88	5	0.719	18.57	5	**0.002**
Commodity	23.43	3	< **0.001**	66.26	3	< **0.001**
Storage Time (d)	2.44	1	0.118	0.01	1	0.935
Container	1.87	3	0.599	2.35	3	0.503
Quality Score	0.05	1	0.830	9.04	1	**0.003**
Land Quartile	1.69	3	0.639	2.57	3	0.463
% HH Earners	0.25	1	0.618	1.60	1	0.206

Household was modeled as a random effect (see Table S1). Thresholds for AFB1 detection and illegal status were 1 and 15 μg/kg, respectively.

Commodity type had a highly significant relationship with the odds of both AFB1 detection (p < 0.001) and illegal status (p < 0.001). Compared to the rice reference level in the AFB1 detection and legal status models, maize, groundnut, and pearl millet each had significantly higher odds ratios (Table S1). This finding confirms that rice is a relatively low-risk commodity in this environment, and that the other three commodities are vulnerable to AFB1 accumulation under local conditions. Neither storage time, container type, landholding quartile, nor the percent of wage-earning household residents had significant effects on the likelihood of AFB1 detection or legal status. The qualitative grain quality score was not significantly associated with the odds of AFB1 detection (p = 0.83) but was significantly associated with AFB1 legal status (p = 0.003). For every one-unit increase in quality score, the odds of illegal levels of AFB1 contamination were reduced by 59% (Table S1).

High fumonisin B1 (FB1) detection rates (84% and 91%) and illegal status rates (70% and 71%) were observed in maize and pearl millet, respectively (Fig. 3). The mean FB1 levels for both maize and pearl millet both exceeded the recommended regulatory maximum of 2 μg/g among contaminated samples, at 36 μg/g for maize and 31 μg/g for pearl millet (Fig. 4). We did not observe significant effects of commodity type on the likelihood of detection or illegal status (p > 0.2; Table 3, Table S2). The storage (storage time and grain quality score) and socioeconomic indicators (landholding quartile and percent wage-earning household residents) had no observable effect on the likelihood of FB1 contamination. Unlike AFB1, which was present in a more representative sub-sample of households, FB1 derived from maize and pearl millet was limited to a more niche population (i.e. those growing non-staples) that could have distinct characteristics. This is a possible explanation for our inability to detect relationships between household-level indicators and FB1 contamination status.

**Table 3:** Summary of GLMM results for FB1 detection and illegal status in maize and pearl millet.

	FB1 Detection			FB1 Illegal Status		
	χ^2^	Df	P	χ^2^	Df	P
(Intercept)	0.93	1	0.334	0.17	1	0.679
Commodity	0.32	1	0.570	0.04	1	0.851
Storage Time (d)	1.11	1	0.292	1.52	1	0.218
Quality Score	0.02	1	0.888	0.29	1	0.590
Land Quartile	1.17	3	0.760	1.38	3	0.711
% HH Earners	0.02	1	0.878	0.54	1	0.463

Household and season were modeled as random effects (see Table S2). Thresholds for FB1 detection and illegal status were 0.0076 and 2 μg/g, respectively.

Deoxynivalenol (DON) contamination of wheat samples was not common among collected samples, with only 2.8% (23/832) yielding detectable levels. No samples collected in our survey yielded DON levels above the regulatory limit of 1 μg/g (Fig. 4). The DON detection rate in wheat was negligible across all sampling time points. The highest detection rate (10.5%) at pre-winter fell to 1.8% by the following season, and gradually declined to zero (Fig. 3).

### 3.4 AFB1 status in rice components

To determine how much of the AFB1 burden in unmilled rice was retained in the rice kernel after milling and polishing, we analyzed paired samples of unmilled and polished rice collected from participants’ households. Paired one-sided t-tests comparing AFB1 contamination revealed that there was significantly less AFB1 in polished rice samples than in unmilled rice (p < 0.0001). The 45% detection rate in rice was reduced to 21% after milling and polishing. Among the unmilled-milled rice pairs, mean AFB1 in contaminated unmilled rice samples was 4.2 μg/kg, compared to 2.2 μg/kg in contaminated polished rice samples. Among polished rice samples with detectable AFB1, there was on average 3.8 μg/kg less toxin than in unmilled rice, with mean toxin difference of 82%. The two sampling time points were not significantly different from one another in total AFB1 load, the magnitude of toxin difference, or the rate of change after milling (p > 0.4), suggesting that the reductive effect of milling was consistent across seasons.

Mean AFB1 contents were 2 μg/kg, 12 μg/kg, and 16 μg/kg for kernel, bran, and husk tissues, respectively. Throughout Unnao District and the nearby sites in peri-urban Lucknow, where rice mills were sampled, rice was milled for farmers on an in-kind basis, where millers processed grain in exchange for the right to keep the by-products of the milling process (bran and husk), which were then sold in local markets. As reported by the millers we surveyed, the most common downstream uses for bran included rice bran oil (with widespread use in the biscuit/cookie industry), other human food products (e.g. bran solids), and poultry feed. All three of these applications may result in downstream human and/or animal exposures to AFB1, and further research is recommended to determine whether these potential exposures are an issue of public health concern.

### 3.5 Estimated dietary mycotoxin exposures

#### 3.5.1 Food grain intake

Commodity-wise consumption frequencies and rates are summarized in Table 4 for each village cluster. Rice and wheat flour were consumed by 100% of consumers across all locations. Groundnut and maize were consumed by between 71-100% and 40–84% of consumers, respectively, across villages, with far lower prevalence in Hasanganj than the Bangarmau clusters. Pearl millet flour was the least commonly consumed commodity, with less than 5% of consumers incorporating this into their diet in all localities except Bangarmau-West, where it was consumed by 33% of those surveyed.

**Table 4:** Percentage of consumers eating each commodity and mean *per capita* daily intake, organized by village cluster.

Commodity	Cluster	%	*Per capita* daily intake (g)
Pearl millet flour	Bangarmau-East	2	<1
	Bangarmau-West	33	10
	Hasanganj	3	2
Groundnut (shell off, packet)	Bangarmau-East	0	0
	Bangarmau-West	0	0
	Hasanganj	10	0
Groundnut (shell on)	Bangarmau-East	100	22
	Bangarmau-West	93	32
	Hasanganj	100	3
Maize flour	Bangarmau-East	51	9
	Bangarmau-West	44	16
	Hasanganj	40	8
Polished rice	Bangarmau-East	100	296
	Bangarmau-West	100	138
	Hasanganj	100	263
Wheat flour	Bangarmau-East	100	320
	Bangarmau-West	100	291
	Hasanganj	100	271


Total households surveyed were 55, 80, and 60 for Bangarmau-East, Bangarmau-West, and Hasanganj clusters, respectively.

**Fig. 5:**
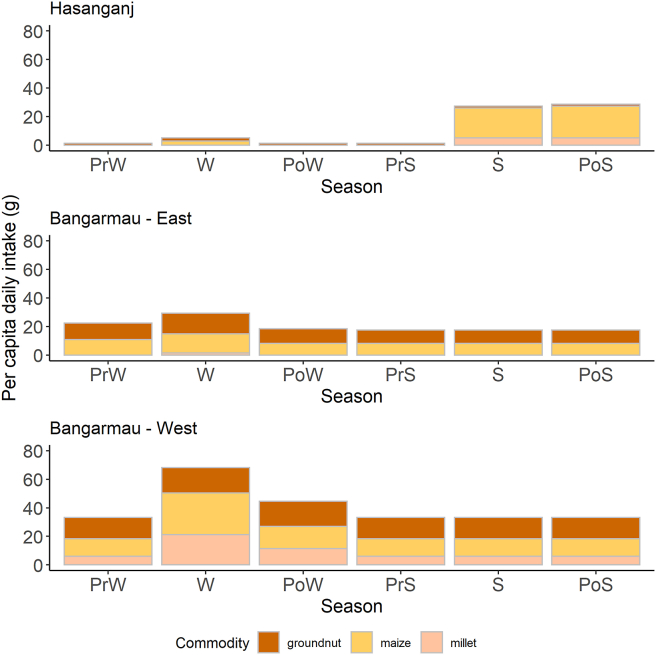
Daily *per capita* non-staple cereals and groundnut consumption (g) across the three village clusters. Seasons: PrW = pre-winter, W = winter, PoW = post-winter, PrS = pre-summer, S = summer, PoS = post-summer. Wheat and rice together constituted ~90% of grain intake by volume and had negligible seasonal, and therefore were omitted from the figure in order to visualize consumption trends in minor commodities.

Daily *per capita* intakes of wheat flour (271–320 g/day) and rice (130–296 g/day) were much higher than for the other commodities across all village clusters and had little seasonal variation. Maize, groundnut, and pearl millet *per capita* intake estimates did not exceed 32 g/day at any time point, but there were strong seasonal trends in consumption (Fig. 5). Consumption of these minor commodities generally peaked in the winter months, except for Hasanganj, where there was a local preference for maize consumption in the summer (June–July). Overall, *per capita* daily cereals and groundnut consumption amounted to between 400 and 600 g across all village clusters and months, with more than 90% of intake attributable to wheat and rice. In Bangarmau-East and Hasanganj, polished rice constituted more than half of total cereal and groundnut intake. Bangarmau-West, which had the most diverse *kharif* season cultivation, had proportionally greater *per capita* consumption of maize, groundnut, and pearl millet than any of the other clusters (Fig. 5).

#### 3.5.2 Mycotoxin exposure estimates

There was substantial seasonal and spatial variation in AFB1 intake, with exposure generally highest in western-most clusters and in the winter months due to cultivation systems and dietary preferences. Maize and groundnuts were the most important contributors of AFB1 in the diet, as expected. These two commodities constituted 96% of *per capita* daily AFB1 intake overall. The range of *per capita* daily AFB1 exposures from a single commodity ranged from zero to 474 ng/kg body weight/day, with most seasonal single-commodity doses less than 20 ng/kg body weight/day. Commodity-wise FB1 exposures from maize and pearl millet were also estimated, revealing moderate doses in some sites and substantial seasonal variation. Daily *per capita* FB1 exposures from a single commodity ranged from zero to 21 μg/kg body weight/day, with maize contributing far more to the dietary FB1 burden than pearl millet. FB1 intake attributable to pearl millet consumption was only observed in the Bangarmau clusters, and was confined to the pre-winter, winter, and post-winter seasons. Maize was a year-round, yet modest, contributor of FB1 to local diets in Bangarmau, with a noticeable peak in the winter season.

**Fig. 6:**
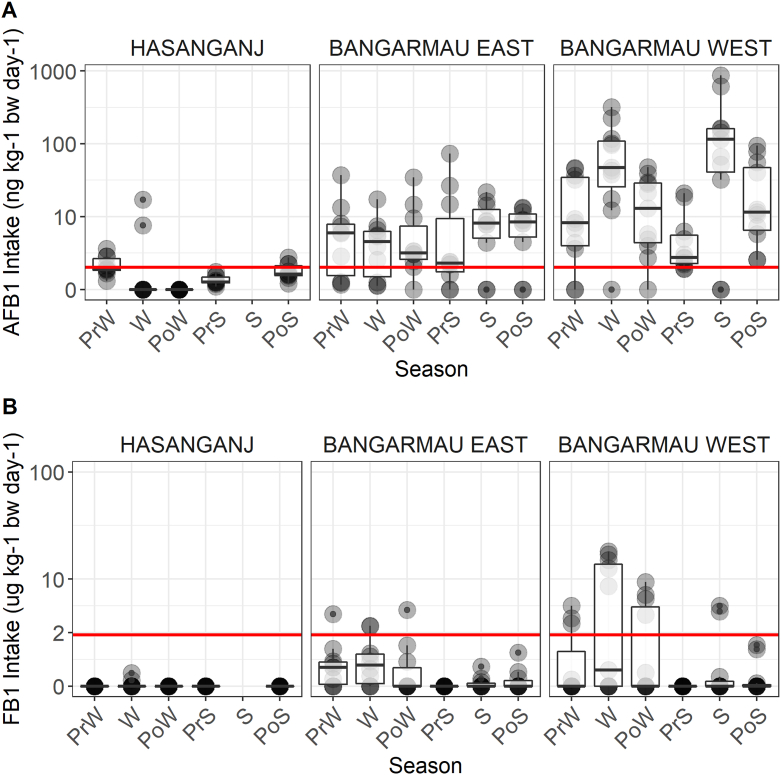
Cumulative *per capita* dietary intake of (A) AFB1 and (B) FB1 across all village clusters. Seasons: PrW = pre-winter, W = winter, PoW = post-winter, PrS = pre-summer, S = summer, PoS = post-summer.

Cumulative *per capita* daily AFB1 and FB1 intakes were computed for each season and locality by summing household *per capita* exposure dose estimates across all commodities. There was substantial spatiotemporal variation in exposure, with the western-most clusters having higher and more frequent exposures (Fig. 6). Average season-wise cumulative *per capita* daily AFB1 intake ranged from 0 to 2.3 ng/kg body weight/day in Hasanganj, 4.0–11.3 ng/kg body weight/day in Bangarmau-East, and 11.9–105.9 ng/kg body weight/day in Bangarmau-West. Averaged across all localities, seasonal *per capita* daily AFB1 intake ranged from 5.4 ng/kg body weight/day (Pre-Summer) to 39.3 ng/kg body weight/day (Summer), with averages in every season exceeding the PMTDI level.

Estimated *per capita* daily FB1 exposures were negligible in Hasanganj, with less than 0.1 μg/kg body weight/day on average in each season (Fig. 6). Levels were higher in the Bangarmau clusters, with between 0 and 0.9 μg/kg body weight/day in Bangarmau East and 0–6.1 in Bangarmau West. We observed a marked decline in FB1 exposure estimates after pre-summer, with household *per capita* intake rarely exceeding the PMTDI level (Fig. 6). Averaged across all localities, mean *per capita* daily FB1 intake ranged from ~0 μg/kg body weight/day in pre-summer to 2.4 μg/kg body weight/day in winter, corresponding to the seasonal consumption patterns for maize and pearl millet.

Our model of cumulative *per capita* daily AFB1 intake revealed a significant effect of season on exposure levels (p < 0.0001), which was consistent with our hypothesis that exposure tracks with seasonal consumption of susceptible commodities (Table 5). A large, significant increase in AFB1 intake during the summer season relative to the pre-winter reference level was observed, which corresponds to high consumption of relatively toxic groundnuts during that season. There was a second exposure peak in the winter season, but we did not find a statistical difference between this season and the reference level (p = 0.14; Table S3). The effect of season was also strongly significant in the FB1 exposure model, with highest exposure in winter (p < 0.001).

**Table 5:** ANOVA output from LMMs for dietary AFB1 and FB1 intake. *P*-values in bold are significant at the p < 0.05 level.

Dietary AFB1 Exposure			
	χ^2^	Df	P
(Intercept)	0.13	1	0.722
Season	18.81	5	**0.002**
Household Size	1.36	1	0.243
**Dietary FB1 Exposure**			
	χ^2^	**Df**	**P**
(Intercept)	0.43	1	0.513
Season	30.02	5	< **0.001**
Household Size	3.92	1	**0.048**

Household, village, and village cluster were modeled as random effects (see Table S3).

Studies from sub-Saharan Africa have shown a negative relationship between household size and AFB1 exposure (Jolly et al., 2006), but we did not detect any significant relationship (p = 0.24) in this context (Table 5). However, we did observe a significant positive effect of household size on FB1 exposure (p = 0.05), suggesting that larger households have higher *per capita* maize consumption. We tested this hypothesis with a simple linear regression model between household size and log-transformed *per capita* maize/millet consumption (g/day) and determined that there was a significant positive relationship (p = 0.02). These findings offer preliminary evidence that mycotoxin exposure doses are substantial in the region and that food system composition plays a major role in determining risk.

## 4 Discussion

This study is among first to profile the temporal dimensions of mycotoxin contamination and dietary exposure at seasonal resolution and offers important insights into risk factors that are suitable targets for community-based participatory research interventions. Our survey of several mycotoxins and the inclusion of all major grain crops present in the study area enabled us to develop a comprehensive understanding of when, where, and how mycotoxins accumulate in this environment. There were distinctive seasonal trends in contamination and estimated dietary exposures, with local food system composition playing a major role in toxin outcomes. As expected, food systems in which maize, groundnuts, and pearl millet were prominent crops had greater detection rates and overall levels of contamination by both AFB1 and FB1 than villages relying largely on rice and wheat as staple foods.

Trends in dietary mycotoxin intake map to locally specific crop production cycles and food preferences, with peak exposures often corresponding to harvest times and/or cultural traditions around the timing of consumption of susceptible commodities. Maize and pearl millet, and to some extent groundnuts were commonly considered winter foods, giving rise to substantial upticks in dietary AFB1 and FB1 exposure during those seasons of increased consumption. Groundnut was heavily contaminated with AFB1 and consumed year-round, leading to a significant spike in AFB1 exposure during the summer months, when contamination levels for this commodity were greatest. As expected, there was a clear relationship between food system composition and toxin exposure, as evidenced by the much lower levels of AFB1 and FB1 intake in Hasanganj compared to the Bangarmau clusters, where susceptible commodities are more prevalent in local diets.

Overall, FB1 levels in both maize and pearl millet were higher than expected, often exceeding regulatory legal limits. While consumption of these commodities was relatively low in Unnao, these levels of contamination may result in FB1 exposures in communities with higher consumption such as the north Indian state of Rajasthan, where pearl millet is a major staple (vom Brocke, 2003). Pearl millet is not typically considered a significant source of fumonisins and aflatoxins, and it has been proposed as a food safety-promoting alternative to maize for its presumed lower risk of contamination (Jurjevic et al., 2005; Vismer et al., 2015). However, it is known that mycotoxin contamination levels can far exceed regulatory threshold levels under conducive conditions (Wilson et al., 2006). It has been shown that year-to-year variation in fungal isolation frequencies is significant in pearl millet, influencing mycotoxigenic potential (Jurjevic et al., 2007). Based on our findings there is evidence that pearl millet could be a significant source of dietary aflatoxin and fumonisin in Indian food systems, and we recommend further investigation in environments where this crop is more prevalent.

DON was of relatively little importance in this food system context, despite the major role of wheat in the local diet. Corroborating earlier evidence, we found that wheat is likely not a major contributor of dietary DON in Unnao. While there have been occasional reports of DON contamination in the Indian context (Mishra et al., 2013), it is yet up for debate whether the fungal pathogen that produces DON, *F. graminearum*, is present in northern India and capable of producing toxins at measurable levels (Backhouse, 2014). In a global survey of mycotoxins in feed, Gruber-Dorninger et al. (2019) found that DON was present in only 23% of South Asian feed samples (n = 1136), with <2% exceeding 0.9 μg/g; by contrast, the same study found that 82% and 69% of samples had detectable AFB1 and fumonisin, respectively. *F. graminearum* is better adapted to temperate climates, so it is possible that the humid sub-tropical climate of Unnao District is not conducive to proliferation of this fungus. Another possible explanation for the low DON contamination levels observed in the area is the relatively high prevalence of preservative amendments in wheat storage. While the utilization of preservatives is still modest in wheat (19% of storage units sampled), it was far more common than in other grains and reflects a greater attention to safety and quality in this commodity.

The duration of storage time emerged as a less important determinant of AFB1 loads in stored grain than expected. Instead, seasonal variations in AFB1 contamination appeared to be largely determined by harvest schedules and perhaps the preferential discharge of better or worse grain earlier in storage time. Hoffmann et al. (2013) observed a similar phenomenon in Kenya, where smallholders allocated highest-contaminated grain for market sale and retained grain with the lowest levels of contamination. Further research into grain usage and decision-making would potentially clarify the extent to which the quality of grain factors into deciding which fractions are destined for household use versus market sale.

Assessment of AFB1 localization across rice components (kernel, bran, and husk) confirmed preferential colonization of the bran and husk by causal fungus *A. flavus*, as has been previously documented in other contexts (Prietto et al., 2015; Purwoko et al., 1991). While rice bran and husk components pose little direct risk to farmers in the form of human food, local utilization of contaminated bran as livestock feed could have detrimental effects for animal nutrition and productivity (Atherstone et al., 2016). Moreover, rice bran is a source of several key nutrients and is emerging as a nutritive ingredient of interest for public health (Borresen & Ryan, 2014), and thus the trade-offs between this product's nutritional qualities and its mycotoxin-related anti-nutritional properties must be considered. Further investigation is needed to evaluate the magnitude and epidemiology of these downstream exposure risks. The fate of rice husks, on the other hand, appears not likely to contribute to downstream dietary exposures.

The *per capita* daily AFB1 intake estimates reported here are in line with previous estimates from India and elsewhere. Murashiki et al. (2017) estimated exposure using similar methods in a maize-consuming region of Zimbabwe and reported probable daily intake of AFB1 between 7.6 and 354.7 ng/kg body weight/day. Liu and Wu (2010) reviewed daily intake estimates (ng/kg body weight/day) reported from several contexts in the developing world: India (4–100), The Gambia (4–115), Ethiopia (1.4–36), Tanzania (0.02–50), Zimbabwe (17.5–42.5), Mexico (14–85), Thailand (53–73), and others. By contrast, aflatoxin exposure rarely exceeds 1 ng/kg body weight/day in European populations (Brera et al., 2015). Our data indicate that daily *per capita* AFB1 intake exceeded the provisional tolerable limit of 1 ng/kg body weight/day in every season on average, but that some localities are substantially more exposed than others, providing evidence of the need for concerted surveillance and targeted action against AFB1 exposures in this region.

To our knowledge, this study is the first to report seasonal estimates of *per capita* daily FB1 exposure in the region. We observed high FB1 detection rates and substantial contamination levels in both maize and pearl millet and across all seasons in which these commodities are prevalent. However, the high contamination levels did not translate to equally concerning dietary exposures, because of the relatively small roles of maize and pearl millet in the Unnao diet. *Per capita* daily exposures exceeded the PMTDI of 2 μg/kg body weight/day in only one locality (Bangarmau-West) and in only one season (winter). The levels of *per capita* daily FB1 intake estimated in our study were below the 2 μg/kg body weight/day PMTDI, and lower than estimates from primarily maize-consuming food systems (Murashiki et al., 2017). Periods with higher mean exposure levels were observed, though, suggesting possible seasonal effects on vulnerable sub-populations such as mothers, infants, and developing fetuses.

It has been demonstrated previously that there is a negative relationship between household size and AFB1 exposure levels in some sub-Saharan African contexts (Jolly et al., 2006), but because of the lower cropping diversity, smaller stored quantities, and shorter storage periods, we hypothesized that there would be an opposite relationship in this study context. We did not observe any significant relationship between household size and AFB1 exposure levels, but there was a significant positive relationship between household size and FB1 exposure, consistent with our hypothesis. This finding is understandable, given the prevalence of relatively non-susceptible staples (rice and wheat) in the region; larger households may have more resources, and thus more opportunity to diversify their cropping systems to include susceptible non-staples such as maize and groundnuts. Whereas increasing dietary diversity in maize-consuming populations has been proposed as an opportunity to reduce exposures (Wu et al., 2014), diversified grain production systems in the Indian context may have greater risk of exposure, and therefore efforts to enhance dietary diversity in the region should acknowledge the importance of food safety and preservation.

The sampling strategy employed in this survey reflected our aim of comprehensively profiling what commodities were being stored, when, where, and under what conditions in the study area. This approach afforded us a rich understanding of the food system dynamics at play across the target locations. However, an analytical disadvantage of this sampling scheme was the inherently unbalanced sample sizes across factor levels. It was infeasible to anticipate or enforce sampling quotas across commodities, locations, and time points. Our use of GLMM, a method well-suited to model unbalanced data (Pardini et al., 2018; Pinheiro & Bates, 2001), enabled sound inferences to be made despite sampling constraints. Another limitation of this study is that it represents only a single year of observations. It is suspected that initial toxin levels upon entering the storage environment, and subsequent post-harvest accumulation, could vary from year to year, dependent on agronomic and meteorological conditions (Munkvold, 2003; Ndemera et al., 2018). The establishment of a multi-year longitudinal data set would enable deeper, more robust, and more generalizable exploration of contamination phenomena.

The dietary exposure assessment presented here constitutes valuable exploratory evidence about mycotoxin intake in the study area, but further investigation is necessary to definitively ascertain local risk levels. The long (12-month) recall period in estimating food consumption in the target communities was not ideal for accurate assessment of food intake (Smith et al., 2014), but was chosen due to resource constraints that prevented more in-depth dietary surveys. Food consumption estimates in Unnao based on shorter recall periods would enhance accuracy and reliability of mycotoxin exposure estimates, and would be necessary to inform local health and nutrition actions. Moreover, because it was not possible to capture detailed intra-household age distributions for use in computing exposures, it is likely that exposure in older children (weighing more than the 20 kg mean body weight used in the calculation) was over-estimated. Further characterization of mycotoxin exposure in the region should aim to capture age distributions more precisely. It is also recommended that exposure estimates based on food contamination be complemented by human biomarker analyses, which can further highlight important risk factors in the local food system.

While yielding several important insights, the present study is only the beginning of a much broader diagnostic process that must explore greater depths in order to contextualize risk factors within inherently heterogenous food systems. A common shortcoming of extant mycotoxin surveillance systems is their inability to translate risk indicators into tractable, meaningful intervention options for farmer communities. We envisage that participatory research could prove useful for delivering meaningful diagnostic and problem-solving options to local contexts in light of the exposure risks identified by this study. Further surveillance efforts combined with participatory technical and behavior change interventions could help resolve unanswered questions about the challenges and opportunities for mycotoxin management in Indian smallholder food systems.

## 5 Conclusions

The findings of this study indicate that the spatiotemporal characteristics of mycotoxin risk factors are highly system-specific, bringing into focus important challenges for agricultural and public health interventions in vulnerable communities. It was determined that mycotoxin accumulation and dietary exposures in smallholder food systems are dynamic across seasons and highly context specific. Contamination levels in the surveyed environment, as well as the consequent dietary intakes, are modulated profoundly by food system composition and dietary preferences. Seasonal and spatial fluctuations in contamination levels and detection rates were notable, illustrating the global need for survey datasets that incorporate sufficient spatial and temporal coverage to allow a meaningful assessment of food system risk. Moreover, this study reveals that food contamination levels are not always reflective of dietary exposure risk; we advocate for co-investigation of contamination status and local dietary consumption patterns, as we have done here, to elucidate the relationships between the food system mycotoxin burden and public health/nutrition risks.

## Funding

This work was supported by the Technical Assistance and Research for Indian Nutrition and Agriculture (TARINA) program of the Tata Cornell Institute for Agriculture and Nutrition. TARINA is funded by the Bill & Melinda Gates Foundation (Grant ID: OPP1137807).

## CRediT authorship contribution statement

**Anthony J. Wenndt:** Conceptualization, Methodology, Investigation, Visualization, Writing – original draft, Software, Data curation, Project administration. **Hari Kishan Sudini:** Methodology, Resources, Writing – review & editing. **Rukshan Mehta:** Methodology, Writing – review & editing. **Prabhu Pingali:** Supervision, Funding acquisition, Writing – review & editing. **Rebecca Nelson:** Conceptualization, Methodology, Writing – review & editing.

## Declaration of competing interest

The authors declare that they have no known competing financial interests or personal relationships that could have appeared to influence the work reported in this paper.

## Figures and Tables

**figs1:**
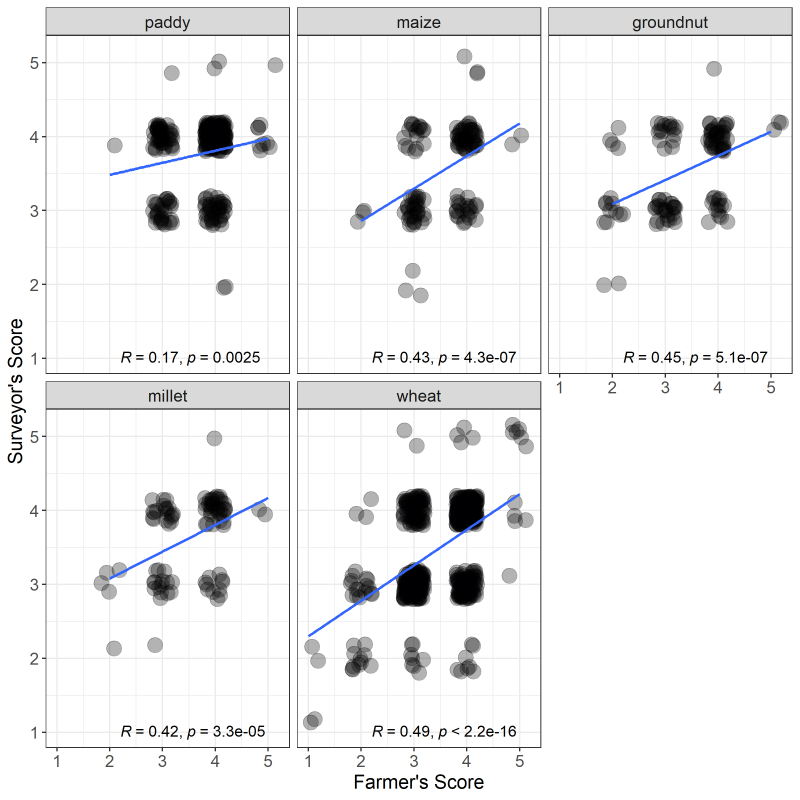
Correlations between farmers’ and surveyors’ grain quality ratings across commodities. Points in the plots are jittered for ease of visualization
