# Supplementary material for: Spatiotemporal assessment of post-harvest mycotoxin contamination in rural North Indian food systems
Source: Food Control. 2021 Aug;126:108071. doi: 10.1016/j.foodcont.2021.108071 (PMC8075802; doi:10.1016/j.foodcont.2021.108071)
Supplement: Multimedia component 1 [file mmc1.docx]

| **Table S1.** GLMM results for AFB1 detection and legal status. Significant p-values (p < 0.05) indicated in bold. | | | | | | |
| --- | --- | --- | --- | --- | --- | --- |
|  | **AFB1 Detected** | | | **AFB1 Illegal** | | |
| *Predictors* | *Odds Ratios* | *CI* | *p* | *Odds Ratios* | *CI* | *p* |
| (Intercept) | 0.62 | 0.11 – 3.58 | 0.590 | 0.19 | 0.02 – 2.40 | 0.201 |
| Season |  |  |  |  |  |  |
| Pre-Winter | Reference |  |  | Reference |  |  |
| Winter | 0.83 | 0.55 – 1.27 | 0.399 | 0.36 | 0.20 – 0.67 | **0.001** |
| Post-Winter | 0.89 | 0.61 – 1.29 | 0.530 | 0.35 | 0.18 – 0.67 | **0.001** |
| Pre-Summer | 0.80 | 0.53 – 1.19 | 0.268 | 1.28 | 0.68 – 2.40 | 0.443 |
| Summer | 1.02 | 0.55 – 1.90 | 0.952 | 3.50 | 1.17 – 10.52 | **0.026** |
| Post-Summer | 1.18 | 0.74 – 1.89 | 0.491 | 1.16 | 0.57 – 2.35 | 0.686 |
| Commodity |  |  |  |  |  |  |
| Paddy | Reference |  |  | Reference |  |  |
| Maize | 0.48 | 0.35 – 0.66 | **<0.001** | 0.10 | 0.04 – 0.22 | **<0.001** |
| Groundnut | 1.32 | 0.92 – 1.89 | 0.130 | 7.90 | 4.70 – 13.26 | **<0.001** |
| Millet | 0.88 | 0.61 – 1.26 | 0.493 | 1.53 | 0.92 – 2.54 | 0.102 |
| Storage Time (d) | 1.00 | 1.00 – 1.00 | 0.118 | 1.00 | 1.00 – 1.00 | 0.935 |
| Storage Container |  |  |  |  |  |  |
| Jute Sack | Reference |  |  | Reference |  |  |
| Poly Sack | 0.88 | 0.56 – 1.40 | 0.598 | 1.33 | 0.65 – 2.71 | 0.432 |
| Other (Mod.) | 0.78 | 0.41 – 1.48 | 0.443 | 0.52 | 0.19 – 1.47 | 0.219 |
| Other (Trad.) | 0.99 | 0.72 – 1.38 | 0.969 | 0.92 | 0.55 – 1.54 | 0.759 |
| Quality Score | 1.05 | 0.69 – 1.58 | 0.833 | 0.41 | 0.23 – 0.74 | **0.003** |
| Land Quartile: |  |  |  |  |  |  |
| Low | Reference |  |  | Reference |  |  |
| Lower-Middle | 1.15 | 0.82 – 1.63 | 0.416 | 1.31 | 0.78 – 2.21 | 0.302 |
| Upper-Middle | 1.04 | 0.74 – 1.47 | 0.804 | 0.93 | 0.55 – 1.58 | 0.788 |
| Upper | 0.99 | 0.69 – 1.43 | 0.973 | 0.66 | 0.35 – 1.23 | 0.189 |
| % HH Earners | 1.00 | 0.99 – 1.01 | 0.618 | 1.01 | 1.00 – 1.02 | 0.206 |
| **Random Effects** | | | | | | |
| σ^2^ | 3.29 | | | 3.29 | | |
| τ_00_ | 0.06 _HHID_ | | | 0.16 _HHID_ | | |
| ICC | 0.02 | | | 0.05 | | |
| N | 135 _HHID_ | | | 135 _HHID_ | | |
| Observations | 541 | | | 541 | | |
| Marginal R^2^ / Conditional R^2^ | 0.101 / 0.118 | | | 0.507 / 0.530 | | |
