# Supplementary material for: Spatiotemporal assessment of post-harvest mycotoxin contamination in rural North Indian food systems
Source: Food Control. 2021 Aug;126:108071. doi: 10.1016/j.foodcont.2021.108071 (PMC8075802; doi:10.1016/j.foodcont.2021.108071)
Supplement: Multimedia component 2 [file mmc2.docx]

| **Table S2.** GLMM results for FB1 detection and legal status. Significant p-values (p < 0.05) indicated in bold. | | | | | | |
| --- | --- | --- | --- | --- | --- | --- |
|  | **FB1 Detected** | | | **FB1 Illegal** | | |
| *Predictors* | *Odds Ratios* | *CI* | *p* | *Odds Ratios* | *CI* | *p* |
| (Intercept) | 8.09 | 0.12 – 560.22 | 0.334 | 1.91 | 0.09 – 40.33 | 0.679 |
| Commodity |  |  |  |  |  |  |
| Millet | Reference |  |  | Reference |  |  |
| Maize | 1.17 | 0.68 – 2.01 | 0.570 | 1.04 | 0.69 – 1.56 | 0.851 |
| Storage Time (d) | 1.00 | 1.00 – 1.01 | 0.287 | 1.00 | 1.00 – 1.01 | 0.055 |
| Quality Score | 1.08 | 0.38 – 3.06 | 0.888 | 1.23 | 0.57 – 2.65 | 0.590 |
| Land Quartile |  |  |  |  |  |  |
| Low | Reference |  |  | Reference |  |  |
| Lower-Middle | 0.94 | 0.37 – 2.35 | 0.888 | 1.34 | 0.66 – 2.73 | 0.415 |
| Upper-Middle | 0.88 | 0.31 – 2.45 | 0.802 | 1.00 | 0.47 – 2.12 | 0.995 |
| Upper | 1.82 | 0.49 – 6.75 | 0.369 | 1.03 | 0.45 – 2.36 | 0.952 |
| % HH Earners | 1.00 | 0.98 – 1.03 | 0.878 | 0.99 | 0.98 – 1.01 | 0.463 |
| **Random Effects** | | | | | | |
| σ^2^ | 3.29 | | | 3.29 | | |
| τ_00_ | 0.62 _HHID_ | | | 0.62 _HHID_ | | |
|  | 0.36 _SEASON_ | | | 0.13 _SEASON_ | | |
| ICC | 0.23 | | | 0.19 | | |
| N | 5 _SEASON_ | | | 5 _SEASON_ | | |
|  | 70 _HHID_ | | | 70 _HHID_ | | |
| Observations | 186 | | | 186 | | |
| Marginal R^2^ / Conditional R^2^ | 0.056 / 0.274 | | | 0.054 / 0.230 | | |
