# Supplementary material for: Spatiotemporal assessment of post-harvest mycotoxin contamination in rural North Indian food systems
Source: Food Control. 2021 Aug;126:108071. doi: 10.1016/j.foodcont.2021.108071 (PMC8075802; doi:10.1016/j.foodcont.2021.108071)
Supplement: Multimedia component 3 [file mmc3.docx]

| **Table S3.** LMM results for the effects of season and household size on AFB1 and FB1 dietary exposures. Significant p-values (p < 0.05) indicated in bold. | | | | | | |
| --- | --- | --- | --- | --- | --- | --- |
|  | **AFB1 intake (ng kg-1 bw day-1)** | | | **FB1 Intake (ug kg-1 bw day-1)** | | |
| *Predictors* | *Estimates* | *CI* | *p* | *Estimates* | *CI* | *p* |
| (Intercept) | -5.50 | -35.79 – 24.78 | 0.722 | -0.51 | -2.03 – 1.01 | 0.513 |
| Season |  |  |  |  |  |  |
| Winter | 14.37 | -4.71 – 33.46 | 0.140 | 1.85 | 0.83 – 2.87 | **<0.001** |
| Post-Winter | -0.41 | -19.50 – 18.67 | 0.966 | 0.38 | -0.64 – 1.41 | 0.461 |
| Pre-Summer | -1.60 | -20.69 – 17.49 | 0.869 | -0.59 | -1.61 – 0.43 | 0.257 |
| Summer | 32.36 | 13.27 – 51.45 | **0.001** | -0.28 | -1.30 – 0.74 | 0.590 |
| Post-Summer | 2.44 | -16.65 – 21.53 | 0.802 | -0.45 | -1.47 – 0.57 | 0.385 |
| HH Size | 1.89 | -1.28 – 5.06 | 0.243 | 0.17 | 0.00 – 0.34 | **0.048** |
| **Random Effects** | | | | | | |
| σ^2^ | 1469.87 | | | 4.21 | | |
| τ_00_ | 245.67 _Cluster:Village:HH_ | | | 0.76 _Cluster:Village:HH_ | | |
|  | 21.96 _Cluster:Village_ | | | 0.00 _Cluster:Village_ | | |
|  | 231.89 _Cluster_ | | | 0.45 _Cluster_ | | |
| ICC | 0.25 | | | 0.22 | | |
| N | 3 _Cluster_ | | | 3 _Cluster_ | | |
|  | 6 _Village_ | | | 6 _Village_ | | |
|  | 31 _HH_ | | | 31 _HH_ | | |
| Observations | 186 | | | 186 | | |
| R^2^_M_ / R^2^_C_ | 0.081 / 0.314 | | | 0.140 / 0.332 | | |
